# The amyloid beta 42/38 ratio as a plasma biomarker of early memory deficits in cognitively unimpaired older adults

**DOI:** 10.1016/j.neurobiolaging.2024.08.009

**Published:** 2024-09-03

**Authors:** Alison R. Bamford, Jenna N. Adams, Soyun Kim, Liv C. McMillan, Rond Malhas, Mark Mapstone, Brian D. Hitt, Michael A. Yassa, Elizabeth A. Thomas

**Affiliations:** a Department of Neurobiology and Behavior, University of California Irvine, Irvine, CA, USA; b Institute for Interdisciplinary Salivary Bioscience Research, University of California Irvine, Irvine, CA, USA; c Center for the Neurobiology of Learning and Memory, University of California, Irvine, Irvine, CA, USA; d Department of Neurology, School of Medicine, University of California Irvine, Irvine, CA, USA

**Keywords:** Alzheimer’s disease, Amyloid-beta, Neurodegeneration, Plasma, Biomarker, Delayed recall

## Abstract

The amyloid beta (Aβ) 42/40 ratio has been widely studied as a biomarker in Alzheimer’s disease (AD); however, other Aβ peptides could also represent relevant biomarkers. We measured levels of Aβ38/40/42 in plasma samples from cognitively-unimpaired older adults and determined the relationships between Aβ levels and amyloid positron-emission-tomography (PET) and performance on a learning and memory task. We found that all Aβ peptides individually and the Aβ42/40 ratio, but not the Aβ42/38 ratio, were significantly correlated with brain amyloid (Aβ-PET). Multiple linear modeling, adjusting for age, sex, education, *APOE4* and Aβ-PET showed significant associations between the Aβ42/38 ratio and memory. Further, associations between the Aβ42/38 ratio and learning scores were stronger in males and in Aβ-PET-negative individuals. In contrast, no significant associations were detected between the Aβ42/40 ratio and any learning measure. These studies implicate the Aβ42/38 ratio as a biomarker to assess early memory deficits and underscore the utility of the Aβ38 fragment as an important biomarker in the AD field.

## 1. Introduction

The accumulation of neuritic plaques containing amyloid beta (Aβ) is a fundamental neuropathological feature of Alzheimer’s disease (AD). Brain Aβ originates from cleavage of the 695 amino acid transmembrane amyloid precursor protein (APP) in a sequential process involving β-secretase and γ-secretase enzymes (Wilquet and De Strooper, 2004, Portelius et al., 2011). The resulting Aβ peptides are comprised of a heterogeneous group of C-terminal peptides ranging in size from 37 to 49 residues due to alternative cleavages of APP of which Aβ37, Aβ38 and Aβ39 represent terminally cleaved products (Matsumura et al., 2014). Past studies have found that Aβ40 is the most abundant amyloid form in the brain and cerebrospinal fluid (CSF), although some studies have shown that Aβ38 is as high, or higher, than Aβ40 in the CSF of AD patients (Wiltfang et al., 2002, Maddalena et al., 2004). Other studies using mass spectrometry have shown that Aβ40 is present in CSF of normal individuals, and those with different neurological conditions, at levels two to three-fold higher than that of Aβ38, while levels of Aβ42 were roughly six to 10-fold lower than Aβ40 (Lame et al., 2011, Wirths and Zampar, 2019, Seino et al., 2021). Nonetheless, Aβ42 is the most widely studied peptide and the focus of most biofluid studies to date, given that Aβ42 exhibits the highest tendency to form aggregates and is the primary constituent in neuritic plaques.

Levels of Aβ42 in the CSF are an established biomarker of AD (Blennow et al., 2015), whereby decreased CSF Aβ42 levels are observed in the CSF of AD patients compared to healthy aged controls (Blennow et al., 2010), likely reflecting aggregation and deposition of Aβ into plaques in brain tissue. However, the Aβ42/40 ratio in the CSF is thought to more accurately reflect changes in Aβ metabolism in AD patients compared to Aβ42 alone, as it corrects for individual baseline differences in both high and low amyloid-producing individuals, and can account for variations in Aβ42 measurements across different platforms (Wiltfang et al., 2007). Similar results have been shown in blood, where plasma and CSF levels of the Aβ42/40 ratios are significantly positively correlated (Janelidze et al., 2021); in this context, several large studies have consistently reported that lower Aβ42/40 ratios in plasma are associated with higher risk of mild cognitive impairment and AD (van Oijen et al., 2006, Graff-Radford et al., 2007, Abdullah et al., 2009, Lambert et al., 2009, Chouraki et al., 2015), greater cognitive decline in healthy control participants at follow-up (Yaffe et al., 2011, Burnham et al., 2020) and that plasma Aβ42/40 ratios are associated with brain amyloid burden, as detected by positron emission tomography (PET) (Nakamura et al., 2018, Schindler et al., 2019).

While studies on Aβ40, Aβ42 and the Aβ42/40 ratio dominate the literature, much less has been reported about the smaller amyloid fragments, namely Aβ38. Previous studies have shown that the Aβ38 peptide does not exhibit toxicity *in vivo*, nor does it accumulate into plaques after overexpression in mice, and can even protect against Aβ42-associated dysfunction (Moore et al., 2018). Interestingly, other studies have suggested that the CSF Aβ42/38 ratio, along with the Aβ42/40 ratio, is a better diagnostic marker of Alzheimer disease than Aβ42 alone (Janelidze et al., 2016).

In this study, we quantified Aβ42, Aβ40 and Aβ38 in plasma samples from cognitively unimpaired older adults and investigated associations between plasma Aβ species, as well as the Aβ42/40 and Aβ42/38 ratios, and PET amyloid burden and a verbal memory task. We aimed to determine if plasma measurements of the Aβ38 peptide or the Aβ42/38 ratio could provide additional information about early cognitive deficits or brain amyloid accumulation over and above the standard Aβ42 or Aβ42/40 ratio.

## 2. Methods

### 2.1. Participants

Participants were recruited from the Biomarker Exploration in Aging, Cognition, and Neurodegeneration (BEACoN) Study at the University of California, Irvine (PI, Yassa). Inclusion criteria consisted of fluency in spoken English, visual and auditory acuity adequate to complete cognitive assessments, and generally normal cognition, defined by the Clinical Dementia Rating scale (Morris, 1993). Participants were excluded if they had a history of significant co-morbid neurological or psychiatric conditions, major medical conditions that significantly affect cognition, a diagnosis of mild cognitive impairment, dementia or other cognitive impairment, or history of alcohol or substance use disorders within the last two years. All experimental protocols were approved by the Institutional Review Board (IRB) of the University of California, Irvine, and all methods were carried out in accordance with relevant guidelines and regulations of the IRB.

### 2.2. Cognitive assessments

Participants completed neuropsychological assessments including the Mini-Mental State Exam (MMSE) (Tombaugh and McIntyre, 1992), the Montreal Cognitive Assessment (MoCA) and the Rey Auditory-Verbal Learning Test (RAVLT) (Schmidt, 1996), a widely-used measure of episodic verbal memory. The RAVLT is a 15-word, multiple-trial, verbal list-learning test that enables assessment of fundamental memory processes, including encoding, learning, retroactive and proactive interference and retrieval. The test consists of 5 learning trials (A1-A5), followed by an immediate recall of a distractor list (B1), then immediate recall of the original list of 15 words (immediate recall, A6). Finally, the participants were tested 20 min later, making up the delayed recall trial (A7). Retroactive interference (RI) was defined by A6/A5 scores.

### 2.3. Neuroimaging

Brain Aβ burden was quantified using ^18^F-florbetapir (FBP) PET imaging on an ECAT High Resolution Research Tomograph (HRRT, CTI/Siemens, Knoxville, TN, USA) as described previously (Adams et al., 2022). Briefly, participants were injected with 10 mCi of tracer and four, 5–min frames were collected from 50 to 70 min post-injection. Data were reconstructed with attenuation correction, scatter correction, and 2 mm^3^ Gaussian smoothing. Additional smoothing was applied to reach an effective resolution of 8 mm^3^. Data were normalized by a whole cerebellum reference region to produce standardized uptake value ratio (SUVR) images. The mean SUVR of a previously validated global cortical composite region was quantified (i.e. global FBP SUVR) and used to determine Aβ-PET positivity [Aβ-PET (+) or Aβ-PET (−)] using a threshold of > 1.11 SUVR (Landau et al., 2012, Landau et al., 2013). Secondary analyses assessed mean FBP SUVRs in composite brain regions, which included the following: frontal cortex (consisting of caudal middle frontal, lateral orbitofrontal, medial orbitofrontal, pars opercularis, pars orbitalis, pars triangularis, rostral middle frontal, superior frontal and frontal pole), parietal cortex (consisting of inferior parietal, precuneus, superior parietal and supramarginal), anterior cingulate cortex (consisting of rostral anterior cingulate, and caudal anterior cingulate), posterior cingulate cortex (consisting of isthmus cingulate, and posterior cingulate) and temporal cortex (consisting of middle temporal and superior temporal regions).

Participants also underwent structural magnetic resonance imaging (MRI) on a 3 T Prisma scanner (Siemens Medical Systems) according to previous studies (Adams et al., 2022) and hippocampal volumetric data was as described previously (Adams et al., 2022).

### 2.4. Plasma collection

All participants gave written informed consent for their blood samples to be used for research. Blood was collected without regard to prandial state, time of day or medication timing. Prior to the PET scan, blood was drawn via venipuncture from each participant into 7 mL lavender top EDTA tubes (BD 366450). Immediately after collection, each tube was gently mixed by inverting 8–10 times to ensure proper mixing of blood and anticoagulant, and then placed on wet ice. Blood samples were centrifuged in a swinging rotor bucket within 1 h of collection at 2600 × RPM at 20°C for 10 min. The isolated plasma was transferred and pooled into a sterile 50 mL polypropylene conical tube and mixed by inversion a few times. The plasma samples were aliquoted by 0.750 mL increments into 2 mL polypropylene cryovials. The plasma aliquots were transferred into a −80 °C freezer for storage until required for analysis.

### 2.5. APOE genotyping

Genotyping was carried out on DNA isolated from saliva samples from BEACoN participants. *APOE* genotypes were determined by a single nucleotide polymorphism (SNP) allelic discrimination assay using Taqman probes to the two *APOE*-defining SNPs, rs429358 (C_3084793_20) and rs7412 (C_904973_10) (ThermoFisher) and these were used to identify *APOE* ε2, ε3, and ε4 alleles. *APOE* genotypes were coded according to the presence (1) or absence (0) of any ε4 allele.

### 2.6. Plasma Aβ quantification

Levels of Aβ42, Aβ40 and Aβ38 were quantified in plasma samples from BEACoN participants using the V-PLEX Aβ Peptide Panel 1 (6E10) Kit 3-plex ECL immunoassay (Meso Scale Discovery (MSD), Gaithersburg, MD). Assays were run according to MSD manufacturers protocol using plasma samples diluted 1:2 in Diluent 35 (MSD). Samples were assayed after a single thaw to room temperature. On each platform, a single batch of reagents was used for all samples. Measurements were performed in duplicate, and sample measurements accepted if coefficients of variation across duplicates were less than 20 %.

### 2.7. Statistical analyses

All statistical analyses were performed using RStudio R 4.3.1, IBM SPSS Software or contchart.com. Raw data were first tested for normality using the Shapiro-Wilk normality test. Data were not normally distributed, hence associations to age and sex were carried out using Spearman correlation analysis and Mann-Whitney U tests, respectively. An outlier analysis was performed using Iglewicz and Hoaglin’s robust test for multiple outliers (two-sided test, modified Z score ≥ 3.5) using Ln-transformed data. The individual values for Aβ38, Aβ40 and Aβ42 from three participants were omitted from further analysis, however no outliers were omitted for the Aβ42/38 ratios. Partial correlations relating Aβ data to global FBP SUVR, hippocampal volume or RAVLT measures were carried out using a non-parametric adjustment and were covaried for age, sex and years education. A Bonferroni correction was applied to the results from our partial correlations analysis to adjust for multiple comparisons. For multiple linear regression models, one assumption is that the residuals must be normally distributed; therefore, we Ln-transformed the data to achieve normal distribution of the residuals. Multiple linear regression modeling predicting learning trial scores and delayed recall scores included age, sex, years education, *APOE4* gene status and global FBP SUVR as covariables. Three linear regression models predicting A5 and A7 were constructed with demographic variables in the first (age, sex, education) (Model 1), the addition of global FBP SUVR in the second (Model 2), and the addition of the Ab42/38 ratio in the third (Model 3).

## 3. Results

### 3.1. Participants

This study involved sixty-nine cognitively unimpaired older adults with an average age of 69.6 yrs +/− 6.5 yrs (range 61–86 years), a majority female (56.5 %) and predominantly white (88.4 %). The mean MMSE for this cohort was 28.6 and the mean MoCA score was 27.4. Full demographic information of the sample is presented in Table 1. Plasma levels of Aβ42 and Aβ40 were detected in 100 % of the participants, while plasma Aβ38 was detected in 81.1 % of participants (Table 2). Despite the lower detection rate overall, the mean level of Aβ38 was higher in plasma than that observed for Aβ40, which is typically thought to represent the most abundant Aβ peptide (Table 2). Only Aβ40 showed a significant correlation with age (Table 2), and no Aβ peptides were correlated with years of education, nor showed sex differences.

### 3.2. Aβ peptides, Aβ-PET, and hippocampal volume

Plasma levels of Aβ42, Aβ40 and the Aβ42/40 ratio were significantly negatively correlated with the global FBP SUVR, in cognitively unimpaired individuals, as determined by partial correlations, adjusting for age and sex (Table 3). Additionally, we found that Aβ38, but not the Aβ42/38 ratio, was negatively correlated with the global FBP SUVRs (Table 3). Regional FBP SUVRs obtained from composite brain regions (frontal, parietal, anterior cingulate, posterior cingulate, temporal composites) showed stronger correlations to Aβ peptides compared to the global FBP SUVR (Suppl Table 1). For example, the strongest negative correlation between any Aβ peptides and the FBP SUVRs was observed for Aβ42 in the temporal cortex (r=−0422; p=0.001; Suppl. Table 1). The only statistically significant correlation observed between the Aβ42/38 ratio and Aβ-PET in any brain region was a marginally positive correlation to the FBP SUVR signal in the temporal cortex (r=0.294; p=0.045; Suppl. Table 1).

We also carried out partial correlation analysis to identify any associations between Aβ species and hippocampal volume, adjusting for age and sex. No significant correlations were detected between any Aβ species, nor any Aβ ratio, and hippocampal volume, considering left, right and bilateral hippocampal volumes (data not shown).

### 3.3. Aβ42/38 ratio and RAVLT

We next assessed the relationship between plasma Aβ peptides, the Aβ42/40 ratio and the Aβ42/38 ratio and verbal memory using partial correlation analysis, correcting for age, sex and years of education, assessing all RAVLT components. Only the Aβ42/38 ratio was significantly correlated with several RAVLT features; these included learning trial A5, immediate and delayed recall (A6 and A7 trials, respectively), the learning slope and RI (Table 4).

**Fig. 1.**
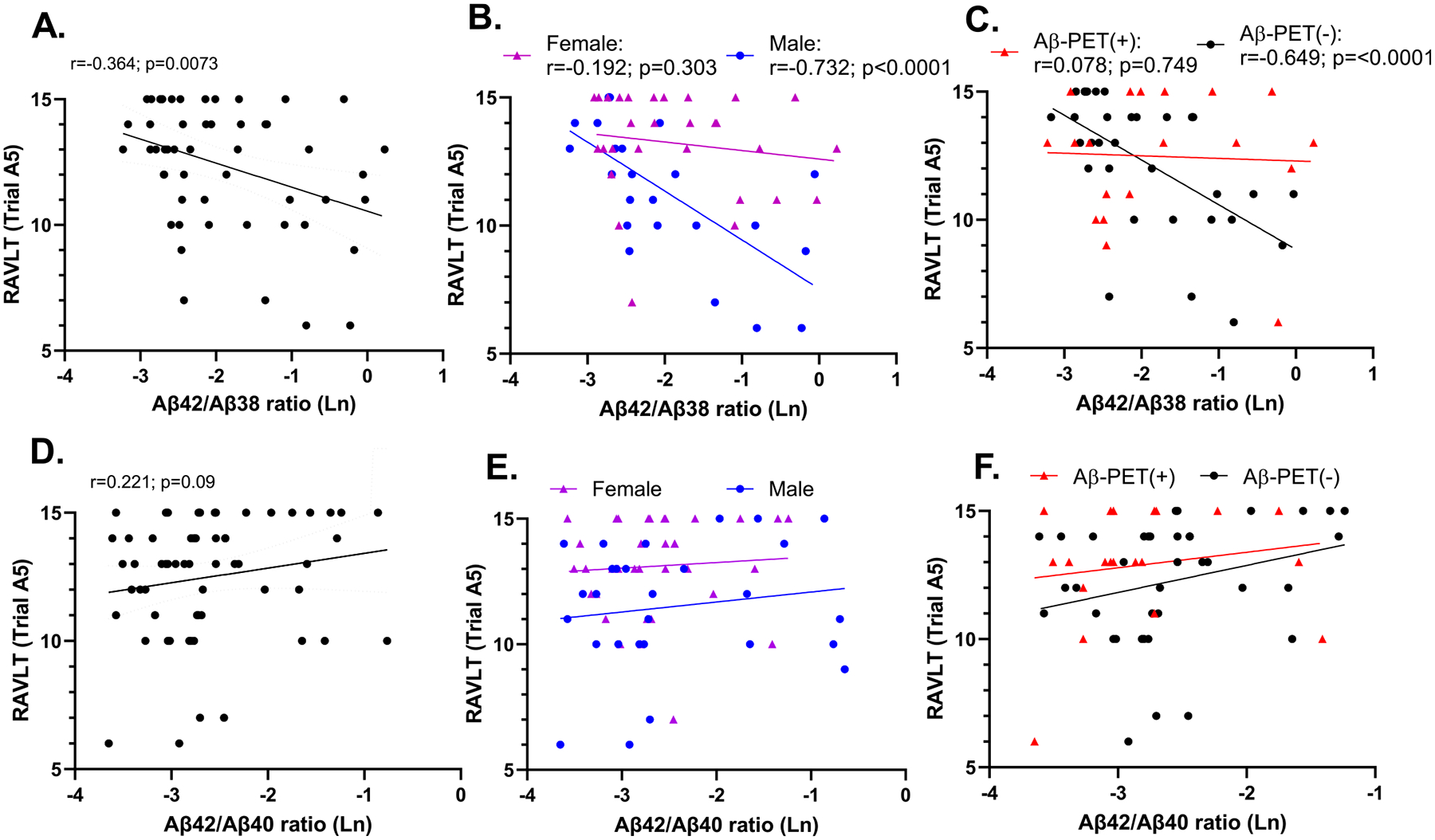
Correlations between the Ab42/38 ratio (A-C) and the Ab42/40 ratio (D-F) and RAVLT verbal learning. Correlations shown reflect unadjusted Pearson or Spearman correlations. Panels B and E depict associations stratified by sex, while C and F depict differences according to Aβ-PET positivity.

Further focusing on learning trial A5 and delayed recall (trial A7), we carried out multiple linear regression analysis to examine the relationship between the Aβ42/38 ratio and memory performance, considering age, sex, education, *APOE4* gene status and brain amyloid status (Aβ-PET (+) vs. Aβ-PET (−)) Out of the demographic variables, we found that only sex was significantly associated with learning trial A5 (b = 7.468; p = 9.27E-04; Table 5) and delayed recall (b = 6.11; p = 0.019; Table 6) with females showing a higher performance on these memory tasks (see also Table 1). Aβ-PET status independently predicted learning (b = 3.842; p = 0.0072; Table 5), but not delayed recall (b = 2.730; p = 0.272; Table 6). The Aβ42/38 ratio strongly negatively predicted performance on both learning (b = −3.343; p = 1.34E-06; Table 5) and delayed recall (b = −3.391; p =2.85E-03; Table 6). Testing for interactions, we found a significant sex by Aβ42/38 ratio interaction (b = 2.08; p = 0.003; Table 5), with males showing a much stronger correlation to learning compared to females (Fig 1) and a significant Aβ-PET by Aβ42/38 ratio interaction (b = 1.897; p = 0.0049; Table 5), where stronger correlations were detected in those individuals considered Aβ-PET (−) (Fig 1). *APOE4* gene status had no effect on any RAVLT measure, nor represented a significant interaction in any model, hence, was not included in the final models.

By comparison, there was no significant correlations between memory performance and the Aβ42/40 ratio, nor any differences between males and females, nor Aβ-PET (+) vs. (−) participants (Table 4; Fig 1). Comparing the Aβ42/40 and Aβ42/38 memory correlations using a Steiger’s z test, revealed a significant difference between these two different ratios (t =−2.221; p< 0.0317), despite the ratios themselves being correlated with one another.

## 4. Discussion

In this study, we show that the plasma Aβ42/38 ratio, but not the commonly-used Aβ42/40 ratio, is significantly associated with memory performance in cognitively-normal, older adults. Aβ accumulation in the brain is well-established toxic event in the pathogenesis of AD (Bateman et al., 2012); however, the predominant focus on the Aβ40/42 ratio in biomarker studies is likely an oversimplified view. A consideration of other Aβ catabolites, especially shorter peptide chain residues, or different proportions of Aβ species, might be more informative regarding the various aspects of AD-related pathology and cognitive decline.

Specifically, we found that the Aβ42/38 ratio is negatively associated with RAVLT learning trials and recall of verbal information in cognitively unimpaired, older individuals, prior to overt decline in MMSE or MoCA scores. The negative direction of the association suggests that higher levels of Aβ38 (i.e. lower Aβ42/38 ratios) are associated with less severe or slower cognitive decline, thereby supporting a protective role for the Aβ38 peptide. Previous studies in mouse models and *in vitro* have shown that the Aβ38 peptide is not toxic and can even protect against Aβ42-associated dysfunction (Moore et al., 2018, Braun et al., 2022). Studies in humans have also suggested a protective role for Aβ38. In a recent study, it was shown that higher CSF levels of Aβ38 were associated with slower decline in cognition (assessed by the MMSE score) and with lower risk of conversion to AD dementia in participants in the BioFINDER cohort (Cullen et al., 2022). These findings were also replicated in the AD Neuroimaging Initiative (ADNI) cohort, whereby higher Aβ38 levels were associated with less decline in MMSE score, but not risk of conversion to AD dementia (Cullen et al., 2022).

Of note, while the relative abundance of the median levels of the amyloid peptides in this study is generally consistent with previous studies in plasma and CSF (Maler et al., 2007, Lame et al., 2011, Shahpasand-Kroner et al., 2018, Seino et al., 2021), the mean plasma level of Aβ38 was found to be higher than Aβ40, which has not been observed in past studies. The discrepancy could be due to several factors, including methodology (i.e. the presence of matrix components and other amyloid binding proteins that may interfere with detection), the greater complexity of Aβ peptides found in plasma (Maler et al., 2007) or different neurological conditions of the participants in past studies.

Although we did not observe any associations with individual Aβ peptides and RAVLT performance, we found notably differences between the Aβ42/Aβ40 and Aβ42/Aβ38 ratios and their relationship to RAVLT measures. This finding could suggest that the proportions of Aβ species and/or relative abundances, are important and preferentially associated with different stages of the disease process. The proteolytic cleavage of the APP glycoprotein results in two product lines generated by the sequential cleavage of three amino acids: product line 1 consists of Aβ49 > Aβ46 > Aβ43 > Aβ40, while product line 2 contains Aβ48 > Aβ45 > Aβ42 (Matsumura et al., 2014). Matsumua et al. found that both Aβ43 and Aβ42 can be terminally cleaved to Aβ38, via the release of a pentapeptide or tetrapeptide, respectively (Matsumura et al., 2014). Hence, one explanation of our findings is that, early in a pathological process, cleavage patterns may shift away from terminal cleavage leading to less Aβ38 and more Aβ42, an increase in Aβ42/Aβ38 ratio that correlates with lower RAVLT scores. Later in the disease process, plaque formation may result in a decrease of serum Aβ42, leading to a net downward trend in the Aβ42/Aβ38 ratio as brain amyloid PET increases. This could explain why the Aβ42/Aβ38 ratio correlated with deficits in RAVLT performance, an early sign of cognitive problems.

Accordingly, we found that plasma levels of Aβ42, Aβ40 and the Aβ42/40 ratio, but not the Aβ42/38 ratio, were significantly negatively correlated with Aβ-PET imaging values in cognitively unimpaired individuals. This is consistent with a recent report, where negative correlations between Aβ42 and the Aβ42/40 ratio and Aβ-PET data were observed in plasma samples from non-demented participants and from individuals across the AD continuum (i.e. when MCI and AD diagnoses are included)(Chatterjee et al., 2023). These results from plasma mirror what is commonly observed in CSF samples (Keshavan et al., 2020).

Interestingly, there were no significant correlations between Aβ-PET and any feature of the RAVLT, when assessed independently, suggesting that measures of brain amyloid levels may not be associated with memory performance. However, when included as an interaction variable in our multiple linear modeling, did we observe a significant Aβ-PET by Aβ42/38 ratio interaction, whereby stronger correlations were detected in those individuals considered to be Aβ-PET (−) compared to Aβ-PET (+). In fact, much of the overall correlation between Aβ42/38 and the A5 learning trial appears to be driven mainly by Aβ-PET (−) individuals. One possibility is that the Aβ42/38 ratio might be a sensitive biomarker for cognitive deficits occurring prior to any substantial accumulation of brain amyloid, although in this cross-sectional study, we cannot know this for sure whether any of the participants will go one to develop amyloid plaques. Alternatively, it is possible that the Aβ42/38 ratio is an early biomarker of non-AD conditions, being associated with cognition but not with AD pathology. We anticipate that future longitudinal studies will be able to shed light on this question.

While not the primary aim of the study, we also found that women performed better on several measures of the RAVLT compared to men. Although initial reviews of the RAVLT literature reported that performance was most significantly influenced by age and years of formal education, and less so by sex (Schmidt, 1996), other studies have reported notable differences between males and females in several aspects of verbal memory tasks. For example, an older study by Geffen and colleagues reported better performance in female adults compared to males in the age range of 16–86 years(Geffen et al., 1990). Additionally, other studies have reported that female participants perform better than men only on the recall trials, but not the recognition trials (Geffen et al., 1990, Miatton et al., 2004); however more recent studies have reported better performance by females on the recognition trials in addition to recall trials (Harris et al., 2002, Van der Elst et al., 2005). Our findings also support a robust sex difference in several aspects of the RAVLT. Further, our linear modeling also revealed a sex interaction for the Aβ42/38 ratio and memory acquisition in the RAVLT, whereby a much stronger negative correlation was observed in male participants compared to females. Given the worse performance overall by males compared to females, this could suggest that the Aβ42/38 ratio is more sensitive to memory deficits in those with lower verbal memory scores initially.

## 5. Conclusions

In summary, we have demonstrated that the Aβ42/38 ratio, but not the widely-utilized Aβ42/40 ratio, nor Aβ42 alone, is associated with early memory deficits in non-demented older individuals, and that these associations occurred independently of amyloid plaque formation. In contrast, the Aβ42/40 ratio was not similarly associated with cognitive performance, but was correlated with brain amyloid burden. These studies further suggest that Aβ38 may be a neuroprotective or resilience biomarker, but nonetheless, highlight the value of the much underutilized Aβ38 peptide as an important biomarker in AD and possibly related conditions.

## Supplementary Material

Supplement

## Figures and Tables

**Fig. 2.**
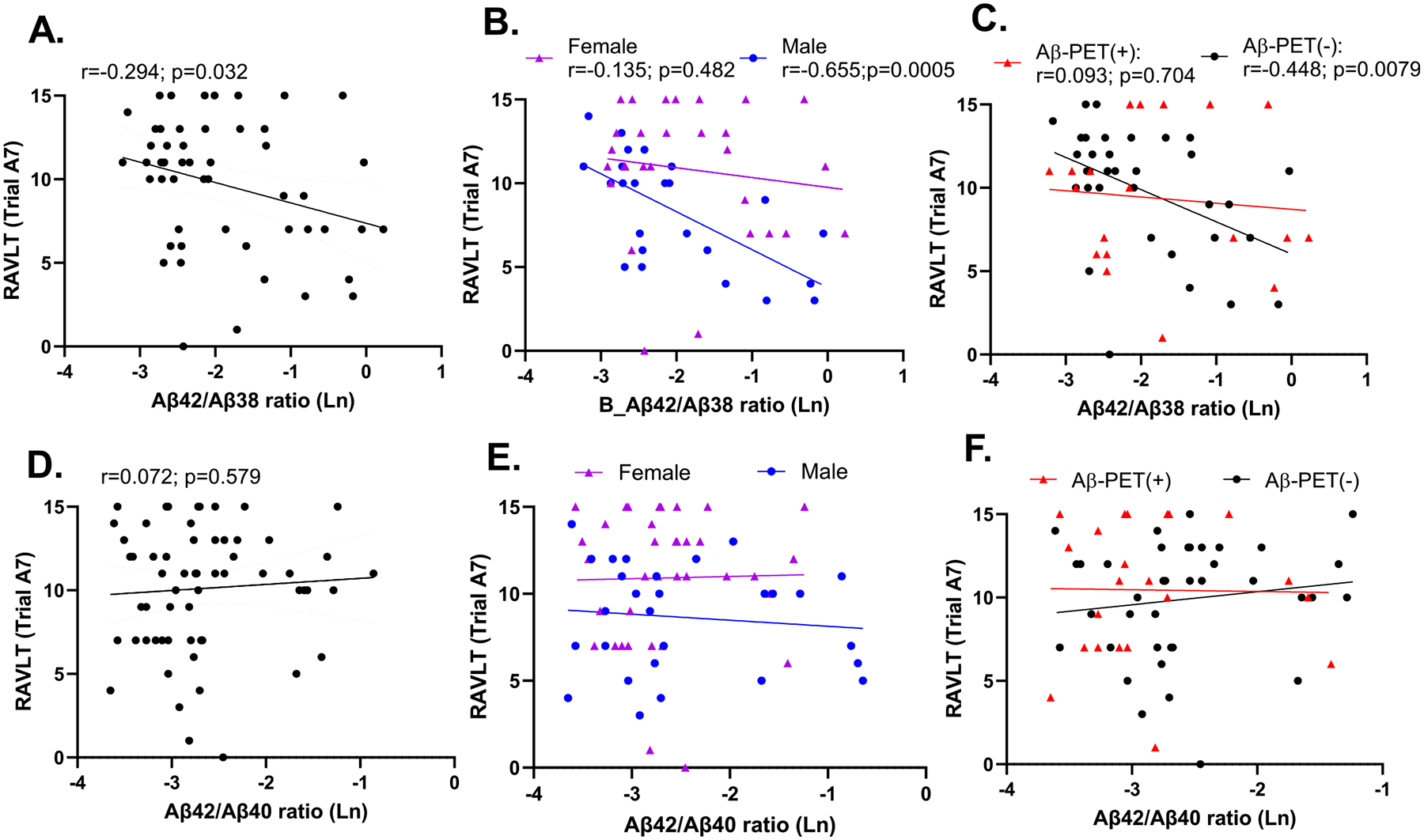
Correlations between the Aβ42/38 ratio (A-C) and the Aβ42/40 ratio (D-F) and RAVLT delayed recall. Correlations shown reflect unadjusted Pearson or Spearman correlations. Panels B and E depict associations stratified by sex, while C and F depict differences according to Aβ-PET positivity.

**Table 1:** Summary of participants used in this study.

	Males:	Females:	Total:	M vs. F
Number	30	39	69	
Mean age in years (range)	69.3 (61–86)	69.9 (62–84)	69.6 (61–86)	n.s.
Mean Edu	17.3	16.2	16.7	n.s.
Mean MMSE	28.3	28.8	28.6	n.s.
Race (White)	90.0 %	87.2 %	88.40 %	n.s.
ApoE4(+)	51.8 % (+)	38.2 % (+)	44.2 %(+)	p=0.234
PET Amyloid (+)	31.0 % (+)	45.9 % (+)	40.9 %(+)	p=0.217
RAVLT-A5 (mean +/− S.D.)	11.5 (2.53)	13.11 (1.91)	12.41 (2.33)	**p=0.005**
RAVLT-A7 (mean +/− S.D.)	8.64 (3.09)	10.92 (3.86)	9.92 (3.7)	**p=0.003**

Edu, Education; MMSE, Mini-mental state examination; ApoE, apolipoprotein E; 18 F-florbetapir PET (positron emission tomography) was used to determine amyloid positivity with a cut-off of 1.11. RAVLT-A5 is the score of the last learning trial. RAVLT-A7 is the delayed recall score. Significant differences between male and females were determined using Mann Whitney tests. n.s. not significant. Significant comparisons are shown in bold.

**Table 2:** Summary of amyloid beta (Aβ) 38, 40 and 42 levels in plasma samples from cognitively unimpaired individuals.

				Age	Sex
Aβ:	N:	Median (range):	Mean ± S.D.:	(r; p-value):	(p-value):
Aβ38	53	115.2 (4.93–2805)	312.2 ± 524.7	0.075; 0.598	0.780
Aβ40	66	188.2 (34.5–462.7)	197.7 ± 71.0	**0.387; 0.001**	0.297
Aβ42	66	10.42 (3.715–185.3)	21.1 ± 30.1	0.214; 0.084	0.847

N=number of samples where the Aβ species measured was above the detection limit, excluding the three high outliers. All samples were above the detection limit for Aβ40 and Aβ42⊡ S.D., standard deviation. Bold font denotes statistically significant correlation using Spearman correlations analysis. Sex associations were determined by Mann-Whitney U test.

**Table 3:** Partial correlations for the association between plasma amyloid beta peptides and brain amyloid burden.

	FBP global SUVR	
Aβ species:	correlation coefficient:	P-value:
Aβ38	−**0.305**	**0.037**
Aβ40	−**0.288**	**0.026**
Aβ42	−**0.353**	0.006*
Aβ42/Aβ40	−**0.296**	**0.022**
Aβ42/Aβ38	0.267	0.07

Brain amyloid burden was determined by use of the 18 F-florbetapir (FBP) global standardized uptake value ratios (SUVR) with a cut-off value of 1.11. Amyloid beta (Aβ). Correlations were adjusted for age and sex. Significant correlations are shown in bold. Asterisk denotes significant finding after Bonferroni correction.

**Table 4:** Partial correlations for the association between amyloid beta peptides and performance on the RAVLT.

		Aβ38	Aβ40	Aβ42	Aβ42/Aβ40	Aβ42/Aβ38
Trial A5	Correlation coefficient	0.249	0.226	0.215	0.147	−**0.425**
	p-value	0.085	0.088	0.106	0.261	0.002*
Trial A6	Correlation coefficient	0.264	0.185	0.14	0.069	−**0.449**
	p-value	0.067	0.163	0.294	0.601	0.001*
Trial A7	Correlation coefficient	0.131	0.047	−0.025	−0.044	−**0.328**
	p-value	0.37	0.724	0.851	0.739	**0.021**
Learning Slope	Correlation coefficient	0.147	−0.006	0.195	0.11	−**0.312**
	p-value	0.314	0.963	0.142	0.405	**0.029**
RI	Correlation coefficient	0.142	0.108	0.056	−0.043	−**0.300**
	p-value	0.329	0.42	0.678	0.742	**0.036**

Partial correlations were adjusted for age, sex, years of education, and were run with a non-parametric adjustment. Learning slope is defined by (A5-A1)/4. Retroactive interference, RI (A6/A5). Asterisk denotes significant finding after Bonferroni correction.

**Table 5:** Multiple linear regression model predicting learning of verbal memory.

Model fit measures:	R^2^:		Adjusted R^2^:			F:		P:
Model 1: Demographics			0.07985			2.794		**0.04809**
	0.1244							
Model 2: Model 1 **+** FBP status			0.0697			2.105		0.09251
	0.1328							
Model 3: Model 2 **+** Aβ42/Aβ38			0.4412			6.302		**5.15E−05**
	0.5245							
Model 3 Results:		Estimate:		SE:	t:		P:	
(Intercept)		11.445		3.589	3.189		**0.0028**	
Age		−0.066		0.044	−1.508		0.1394	
Sex		6.082		1.391	4.373		**8.51E−05**	
Education		−0.138		0.147	−0.937		0.3544	
FBP SUVR		3.842		1.355	2.835		**0.0072**	
Aβ42/Aβ38		−3.343		0.589	−5.678		**1.34E−06**	
Sex:Aβ42/Aβ38		2.086		0.661	3.158		**0.0030**	
FBP:Aβ42/Aβ38		1.897		0.637	2.976		**0.0049**	

Data reflect RAVLT learning trial A5. Demographic data included age, sex and education. SE, standard error. 18 F-florbetapir (FBP) standardized uptake value ratio (SUVR).

**Table 6:** Multiple linear regression model predicting delayed recall.

Model fit measures:	R^2^:	Adjusted R^2^:	F:	p:
Model 1: Demographics	0.1059	0.06044	2.33	0.08357
Model 2: Model 1 **+** FBP status	0.1144	0.05001	1.776	0.1467
Model 3A: Model 2 **+** Aβ42/Aβ38	0.3196	0.2005	2.684	**0.02236**
Model 3 Results:	Estimate:	SE:	t:	p:
(Intercept)	12.37103	6.50591	1.902	0.06446
Age	−0.02641	0.07981	−0.331	0.74245
Sex	6.11206	2.52107	2.424	**0.01995**
Education	−0.52431	0.26664	−1.966	0.05622
FBP status	2.73003	2.45601	1.112	0.27296
Aβ42/Aβ38	−3.39176	1.067	−3.179	**2.85E−03**
Sex:Aβ42/Aβ38	1.99808	1.19722	1.669	0.10294
FBP:Aβ42/Aβ38	1.64444	1.15518	1.424	0.16234

Data reflect RAVLT trial A7. Demographic data included age, sex and education. SE, standard error. 18 F-florbetapir (FBP) standardized uptake value ratio (SUVR).
