# Supplementary material for: The amyloid beta 42/38 ratio as a plasma biomarker of early memory deficits in cognitively unimpaired older adults
Source: Neurobiol Aging. Author manuscript; Available in PMC 2025 Jan 8. (PMC11706698; doi:10.1016/j.neurobiolaging.2024.08.009)
Supplement: Supplement [file NIHMS2041126-supplement-Supplement.pdf]

Suppl. Table 1. Partial Non-parametric correlations for plasma amyloid beta peptides and FBP separated by specific brain regions.

		**Aβ38**	**Aβ40**	**Aβ42**	**Aβ42/Aβ40**	**Aβ42/Aβ38**
FBP mean	Correlation Coefficient	**-0.305**	**-0.288**	**-0.353**	**-0.296**	0.267
	P-value	**0.037**	**0.026**	**0.006**	**0.022**	0.07
FBP frontal	Correlation Coefficient	**-0.328**	**-0.29**	**-0.346**	**-0.296**	0.286
	P-value	**0.025**	**0.025**	**0.007**	**0.022**	0.052
FBP antcingulate	Correlation Coefficient	**-0.306**	**-0.317**	**-0.345**	**-0.272**	0.256
	P-value	**0.037**	**0.014**	**0.007**	**0.036**	0.082
FBP postcingulate	Correlation Coefficient	-0.279	**-0.256**	**-0.279**	-0.25	0.272
	P-value	0.058	**0.048**	**0.031**	0.054	0.064
FBP parietal	Correlation Coefficient	-0.221	**-0.255**	**-0.305**	-0.237	0.194
	P-value	0.136	**0.049**	**0.018**	0.068	0.19
FBP temporal	Correlation Coefficient	**-0.347**	**-0.321**	**-0.411**	**-0.347**	**0.294**
	P-value	**0.017**	**0.012**	**0.001**	**0.007**	**0.045**

18F-florbetapir (FBP) standardized uptake value ratio (SUVR). The unadjusted p-values are significant at $p<0.05$. Bonferroni-adjusted p-values are significant at $p<0.0083$.
